# Analysis of acute COVID-19 including chronic morbidity: protocol for the deep phenotyping National Pandemic Cohort Network in Germany (NAPKON-HAP)

**DOI:** 10.1007/s15010-023-02057-0

**Published:** 2023-07-11

**Authors:** Fridolin Steinbeis, Charlotte Thibeault, Sarah Steinbrecher, Yvonne Ahlgrimm, Ira an Haack, Dietrich August, Beate Balzuweit, Carla Bellinghausen, Sarah Berger, Irina Chaplinskaya-Sobol, Oliver Cornely, Patrick Doeblin, Matthias Endres, Claudia Fink, Carsten Finke, Sandra Frank, Sabine Hanß, Tim Hartung, Johannes Christian Hellmuth, Susanne Herold, Peter Heuschmann, Jan Heyckendorf, Ralf Heyder, Stefan Hippenstiel, Wolfgang Hoffmann, Sebastian Ulrich Kelle, Philipp Knape, Philipp Koehler, Lucie Kretzler, David Manuel Leistner, Jasmin Lienau, Roberto Lorbeer, Bettina Lorenz-Depiereux, Constanze Dorothea Lüttke, Knut Mai, Uta Merle, Lil Antonia Meyer-Arndt, Olga Miljukov, Maximilian Muenchhoff, Moritz Müller-Plathe, Julia Neuhann, Hannelore Neuhauser, Alexandra Nieters, Christian Otte, Daniel Pape, Rafaela Maria Pinto, Christina Pley, Annett Pudszuhn, Philipp Reuken, Siegberg Rieg, Petra Ritter, Gernot Rohde, Maria Rönnefarth, Michael Ruzicka, Jens Schaller, Anne Schmidt, Sein Schmidt, Verena Schwachmeyer, Georg Schwanitz, Werner Seeger, Dana Stahl, Nicole Stobäus, Hans Christian Stubbe, Norbert Suttorp, Bettina Temmesfeld, Sylvia Thun, Paul Triller, Frederik Trinkmann, Istvan Vadasz, Heike Valentin, Maria Vehreschild, Christof von Kalle, Marie von Lilienfeld-Toal, Joachim Weber, Tobias Welte, Christian Wildberg, Robert Wizimirski, Saskia Zvork, Leif Erik Sander, Janne Vehreschild, Thomas Zoller, Florian Kurth, Martin Witzenrath

**Affiliations:** 1 Department of Infectious Diseases, Respiratory Medicine and Critical Care, Charité-Universitätsmedizin Berlin, Corporate Member of Freie Universität Berlin and Humboldt-Universität zu Berlin, Charitéplatz 1, 10117 Berlin, Germany; 2 Berlin Institute of Health at Charité-Universitätsmedizin Berlin, Berlin, Germany; 3 Division of Infectious Diseases, Department of Medicine II, Faculty of Medicine, Medical Centre-University of Freiburg, Freiburg, Germany; 4 Department of Respiratory Medicine/Allergology, Medical Clinic 1, University Hospital Frankfurt, Goethe University Frankfurt, Frankfurt am Main, Germany; 5 Department of Medical Informatics, University Medical Center Göttingen, Göttingen, Germany; 6 Faculty of Medicine, Institute of Translational Research, Cologne Excellence Cluster On Cellular Stress Responses in Aging-Associated Diseases (CECAD), University of Cologne, Cologne, Germany; 7 Faculty of Medicine, Department I of Internal Medicine, Center for Integrated Oncology Aachen Bonn Cologne Duesseldorf (CIO ABCD) and Excellence Center for Medical Mycology (ECMM), University of Cologne, Cologne, Germany; 8 Deutsches Herzzentrum der Charité, Klinik für Kardiologie, Angiologie und Intensivmedizin, Berlin, Germany; 9 Department of Neurology with Experimental Neurology, Charité-Universitätsmedizin Berlin, Corporate Member of Freie Universität Berlin and Humboldt-Universität zu Berlin, Berlin, Germany; 10 Center for Stroke Research Berlin, Charité-Universitätsmedizin Berlin, Corporate Member of Freie Universität Berlin and Humboldt-Universität zu Berlin, Berlin, Germany; 11 Department of Anesthesiology, University Hospital of Ludwig-Maximilians-University (LMU), Munich, Germany; 12 Department of Medicine III, University Hospital of Ludwig-Maximilians-University (LMU), Munich, Germany; 13 COVID-19 Registry of the LMU Munich (CORKUM), University Hospital of Ludwig-Maximilians-University (LMU), Munich, Germany; 14 Department of Medicine V, Internal Medicine, Infectious Diseases and Infection Control, University Hospital Giessen and Marburg, Giessen, Germany; 15 German Center for Lung Research (DZL), Institute of Lung Health (ILH), Excellence Cluster Cardiopulmonary Institute (CPI), Justus Liebig-University, Giessen, Germany; 16 Institute of Clinical Epidemiology and Biometry, University Würzburg, Würzburg, Germany; 17 Clinical Trial Center, Institute for Medical Data Science, University Hospital Würzburg, Würzburg, Germany; 18 Department of Internal Medicine I, University Hospital Schleswig-Holstein, Kiel, Germany; 19 Charité-Universitätsmedizin Berlin, Corporate Member of Freie Universität Berlin and Humboldt-Universität zu Berlin, NUM Coordination Office, Berlin, Germany; 20 Institute for Community Medicine Section Health Care Epidemiology and Community Health, University Medicine Greifswald, Greifswald, Germany; 21 Department of Cardiology, Charité-Universitätsmedizin Berlin, Corporate Member of Freie Universität Berlin and Humboldt-Universität zu Berlin, Berlin, Germany; 22 Department of Cardiology and Angiology, Goethe University Frankfurt, Frankfurt, Germany; 23 Institute of Computer-Assisted Cardiovascular Medicine, Deutsches Herzzentrum der Charité, Berlin, Germany; 24 Department of Radiology, University Hospital of Ludwig-Maximilians-University (LMU), Munich, Germany; 25 Institute of Epidemiology, Helmholtz Center Munich, Munich, Germany; 26 Department of Endocrinology and Metabolism, Charité-Universitätsmedizin Berlin, Corporate Member of Freie Universität Berlin and Humboldt-Universität zu Berlin, Berlin, Germany; 27 German Center for Diabetes Research, Munich-Neuherberg, Germany; 28 Department of Internal Medicine IVM, University Hospital Heidelberg, Heidelberg, Germany; 29 Max Von Pettenkofer Institute and Gene Center, Virology, National Reference Center for Retroviruses, Ludwig-Maximilians-University Munich (LMU), Munich, Germany; 30 German Center for Infection Research (DZIF), Partner Site Munich, Munich, Germany; 31 Department of Epidemiology and Health Monitoring, Robert Koch Institute, Berlin, Germany; 32 Faculty of Medicine, FREEZE-Biobank, Medical Center-University of Freiburg, Freiburg, Germany; 33 Faculty of Medicine, Institute for Immunodeficiency, Medical Center-University of Freiburg, Freiburg, Germany; 34 Department of Psychiatry, Charité-Universitätsmedizin Berlin, Corporate Member of Freie Universität Berlin and Humboldt-Universität zu Berlin, Berlin, Germany; 35 German Center for Cardiovascular Research (DZHK), Partner Site Berlin, Berlin, Germany; 36 Department of ENT, Charité-Universitätsmedizin Berlin, Corporate Member of Freie Universität Berlin and Humboldt-Universität zu Berlin, Berlin, Germany; 37 Department of Internal Medicine IV, University Hospital Jena, Jena, Germany; 38 Max-Planck-Institute for Heart and Lung Research, Bad Nauheim, Germany; 39 Independent Trusted Third Party, University Medicine Greifswald, Greifswald, Germany; 40 Department of Medicine II, University Hospital of Ludwig-Maximilians-University (LMU), Munich, Germany; 41 Department of Pneumology and Critical Care Medicine, Thoraxklinik, Translational Lung Research Center Heidelberg (TLRC), University of Heidelberg, Heidelberg, Germany; 42 Department of Biomedical Informatics, Center for Preventive Medicine and Digital Health Baden-Württemberg (CPD-BW), University Medical Center Mannheim, Heidelberg University, Mannheim, Germany; 43 Department of Internal Medicine, Infectious Diseases, University Hospital Frankfurt, Goethe University Frankfurt, Frankfurt am Main, Germany; 44 Department of Internal Medicine II, Jena University Hospital, Jena, Germany; 45 Leibniz Institute for Natural Product Research and Infection Biology, Hans-Knöll-Institute, Jena, Germany; 46 Medical Department 2, Hematology/Oncology and Infectious Diseases, University Hospital of Frankfurt, Goethe University Frankfurt, Frankfurt, Germany; 47 Department I for Internal Medicine, Faculty of Medicine, University of Cologne, Cologne, Germany

**Keywords:** SARS-CoV-2, COVID-19, Deep phenotyping, Infectious disease, Coronavirus

## Abstract

**Background:**

The severe acute respiratory syndrome corona virus 2 (SARS-CoV-2) pandemic causes a high burden of acute and long-term morbidity and mortality worldwide despite global efforts in containment, prophylaxis, and therapy. With unprecedented speed, the global scientific community has generated pivotal insights into the pathogen and the host response evoked by the infection. However, deeper characterization of the pathophysiology and pathology remains a high priority to reduce morbidity and mortality of coronavirus disease 2019 (COVID-19).

**Methods:**

NAPKON-HAP is a multi‐centered prospective observational study with a long‐term follow‐up phase of up to 36 months post-SARS-CoV-2 infection. It constitutes a central platform for harmonized data and biospecimen for interdisciplinary characterization of acute SARS-CoV-2 infection and long-term outcomes of diverging disease severities of hospitalized patients.

**Results:**

Primary outcome measures include clinical scores and quality of life assessment captured during hospitalization and at outpatient follow-up visits to assess acute and chronic morbidity. Secondary measures include results of biomolecular and immunological investigations and assessment of organ-specific involvement during and post-COVID-19 infection. NAPKON-HAP constitutes a national platform to provide accessibility and usability of the comprehensive data and biospecimen collection to global research.

**Conclusion:**

NAPKON-HAP establishes a platform with standardized high-resolution data and biospecimen collection of hospitalized COVID-19 patients of different disease severities in Germany. With this study, we will add significant scientific insights and provide high-quality data to aid researchers to investigate COVID-19 pathophysiology, pathology, and chronic morbidity.

## Background

In the last 3 years, the global severe acute respiratory syndrome corona virus 2 (SARS-CoV-2) pandemic has caused a high burden of mortality and morbidity worldwide. In December 2019, first cases of a SARS-CoV-2 infection were described in Wuhan, Hubei province, China [1, 2]. As of December 2022, more than 650 M confirmed infections and 6.6 M deaths—with a significant proportion of SARS-CoV-2 infections and deaths suggested not to be reported at all—have been registered officially [3–5]. Over the past 3 years, an astonishing global research effort has yielded a multitude of scientific insights scrutinizing the course of coronavirus disease 2019 (COVID-19) [6]. Deep immunological and molecular characterization of COVID-19 patients has generated crucial insight into the pathophysiology of SARS-CoV-2 infection. Despite these advances, treatment options still remain limited. Effective vaccination, however, has saved lives at an unprecedented scale [7–10].

COVID-19 disease severity ranges from asymptomatic and mild disease to critical illness as defined by WHO [11], and plentiful risk factors have been investigated and assigned to patient outcome [12]. In critical COVID-19 disease, SARS-CoV-2 infection leads to acute respiratory distress syndrome (ARDS) with the need of mechanical ventilation (MV) and sometimes extracorporeal membrane oxygenation (ECMO) [13, 14]. In severe COVID-19 and in mild disease, cardiovascular, neurologic, and further extra-pulmonary complications have been described [15, 16]. Persisting symptoms beyond the acute phase, particularly of the respiratory, cardio-circulatory and neuropsychiatric systems have been described, yet so far not sufficiently characterized.

**Fig. 1:**
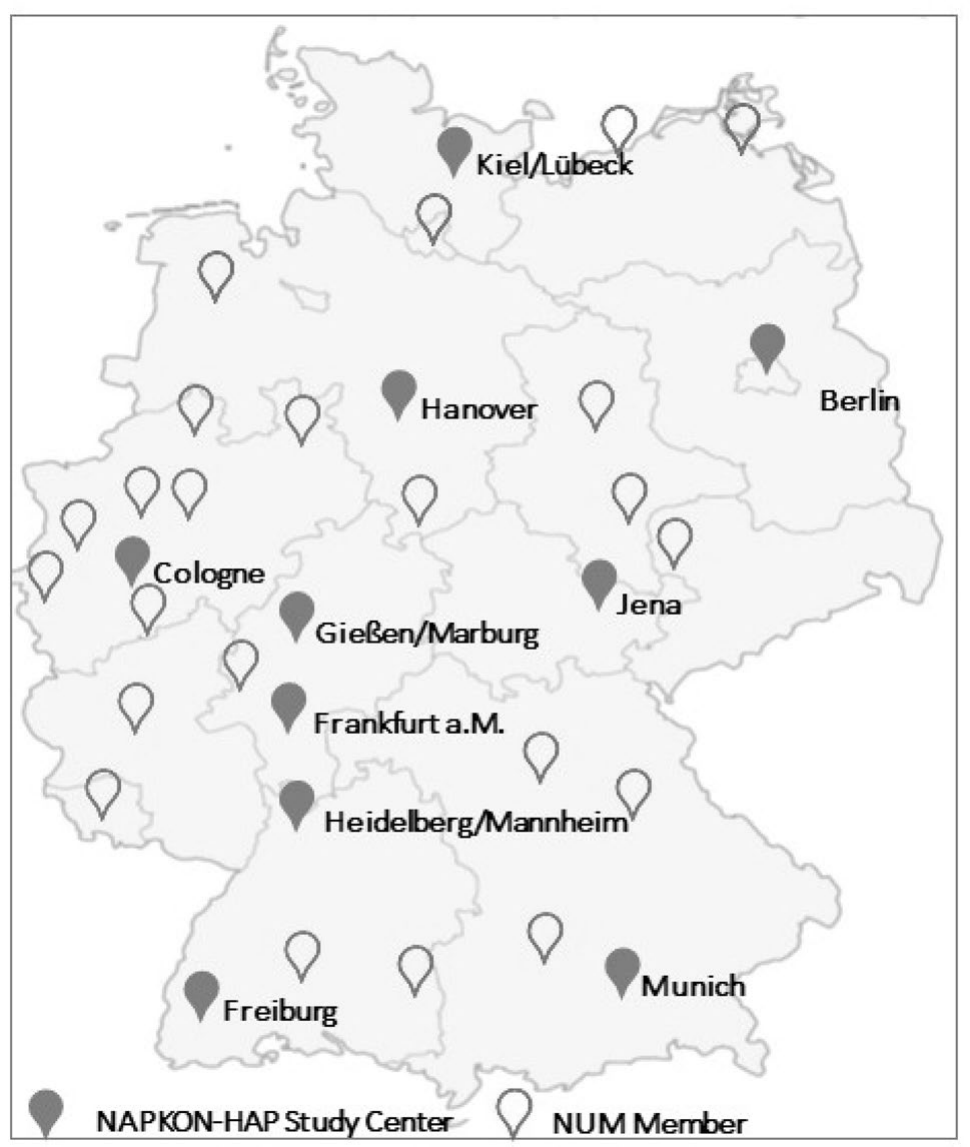
NUM members and NAPKON-HAP study centers in Germany. The following university clinics participate in NAPKON-HAP: Berlin, Cologne, Frankfurt, Freiburg, Gießen, Hannover, Heidelberg, Kiel/Lübeck, Munich (LMU). NUM members (as of November 2020) are Aachen, Augsburg, Berlin, Bielefeld (OWL), Bochum, Bonn, Cologne, Dresden, Düsseldorf, Erlangen, Essen, Frankfurt, Freiburg, Gießen/Marburg, Göttingen, Greifswald, Halle, Hamburg, Hannover, Heidelberg, Jena, Leipzig, Magdeburg, Mainz, Mannheim, Munich (LMU/TU), Münster, Oldenburg, Regensburg, Rostock, Saarland (UKS), Schleswig–Holstein (UKSH), Tübingen, Ulm, Würzburg

A plethora of crucial questions remain unanswered and highlight the urgent need for well-characterized patient cohorts. To spark effective and high-impact research into COVID-19 in Germany, a network of medical universities (NUM), funded by the German Federal Ministry of Education and Research (BMBF), has been established (Fig. 1) in 2020. Within this consortium, a National Pandemic Cohort Network (NAPKON) was developed for three multicenter observational cohort studies including a deep phenotyping platform (high-resolution platform—HAP). Ten university hospitals within Germany participate in the NAPKON-HAP study (Fig. 1).

NAPKON-HAP implements a research infrastructure for high-resolution phenotyping of patients with SARS-CoV-2 infection of different disease severities. The primary objective is to provide a comprehensive and harmonized collection of data and biosamples for researchers and for participation in international research collaborations for the purpose of studying COVID-19 and future pandemics.

The deep phenotyping data and high-quality biospecimen collection provided by NAPKON-HAP enables research into the role of immunological, pulmonary, cardiovascular, neuropsychiatric, and endocrine events in COVID-19, among others. NAPKON-HAP serves to foster research into innate and adaptive immunity, to disseminate targets of SARS-CoV-2-induced innate and adaptive immune responses and its change over time to identify biomarkers for protective immunity and to develop therapeutic strategies and support development of vaccines. We also intend to provide data and biospecimen platform to identify biomarkers for early estimation of disease progression and provide prognostic markers acute and long-term outcome. NAPKON-HAP aims to correlate those findings within a collaborative research effort of immunological, microbiological, and virological expertise to clinical parameters to improve understanding of SARS-CoV-2-induced disease progression and to assess effects of specific COVID-19 treatments.

## Methods

NAPKON (www.napkon.de) provides a platform for harmonized collection and use of data and biospecimens, involving all health sectors within Germany. It ensures centrally coordinated time- and cost-efficient use of resources with high data and biomaterial quality. NAPKON aims to address scientific and health care-relevant questions comprehensively to provide representative, evidence-based information on COVID-19-specific risk factors, disease progression, and long-term sequelae.

NAPKON incorporates three different cohort platforms to represent the wide spectrum of COVID-19 severity and the associated characteristics of patients enrolled at different institutions. The cross-sector platform (NAPKON-SÜP) captures clinically ill patients with COVID-19 through a network of hospitals of all care levels and outpatient practices. The population-based platform (NAPKON-POP) recruits on the basis of public-health authority registries independent of disease severity and surveys representative long-term outcome over time. The high-resolution platform (NAPKON-HAP) comprehensively studies hospitalized patients with SARS-CoV-2 infection and evaluates their organ-specific sequelae after hospital discharge longitudinally and with high granularity.

### Study design of NAPKON-HAP

NAPKON-HAP is a multi‐centered prospective observational study with a long‐term follow‐up phase of up to 36 months post-SARS-CoV-2 infection. The protocol was developed along the standards defined by the German Corona Consensus (GECCO) and evolved from the Berlin prospective COVID‑19 patient cohort (Pa‑COVID‑19) [17, 18]. The protocol was developed in accordance with the standardized protocol for the rapid, coordinated clinical investigation of severe acute infections by pathogens of public-health interest published by the International Severe Acute Respiratory and Emerging Infection Consortium (ISARIC) [19]. NAPKON-HAP is registered at clinicaltrials.gov (NCT04747366).

NAPKON-HAP constitutes a central platform of harmonized data for interdisciplinary characterization of acute SARS-CoV-2 infection and long-term outcomes of diverging disease severities of hospitalized patients. The first patient was enrolled in November 2020 in NAPKON-HAP at Charité Berlin, Germany. As of December 2022, 700 patients have been enrolled, and of those, 600 enrolled with COVID-19 until December 2021. Since then, patients were recruited into the amended NAPKON 2.0, aiming at the implementation of control cohorts (see “Control groups”). The preliminary end date of the study is the date of the last visit of the last patient undergoing the study.

### Study inclusion criteria

Patients hospitalized at one of the participating study sites for COVID-19, confirmed by means of a positive SARS-CoV-2 PCR or initial positive rapid diagnostic test, in conjunction with characteristic radiological findings and infection of the respiratory tract, are eligible for inclusion. During hospital treatment, data are collected longitudinally from patients until discharge. After hospital discharge, structured follow-up visits over a period of up to 36 months after onset of first symptoms of COVID-19 will take place. Inclusion criteria are (i) age of 18 years or older; (ii) written consent to participate in the study by patient or appropriate legal representative; (iii) hospitalization at time of enrollment; (iv) positive SARS-CoV-2 PCR or initial positive rapid diagnostic test with positive PCR in due course, with typical clinical symptoms. Exclusion criteria are refusal to participate by patient or legal representative, or any condition that prohibits supplemental blood sampling.

### Control groups

To distinguish between COVID-19 specific and non-specific findings, control groups of non-COVID-19 community acquired pneumonia (CAP) and non-COVID-19 ARDS were established. Further, to enable investigation into the mechanisms behind immunological failure to develop a protective immune response after SARS-CoV-2 vaccination, a separate group of vaccine break through infections was introduced.

### Patient assessment and biosampling: in-hospital study visits and outpatient follow-up

**Fig. 2:**
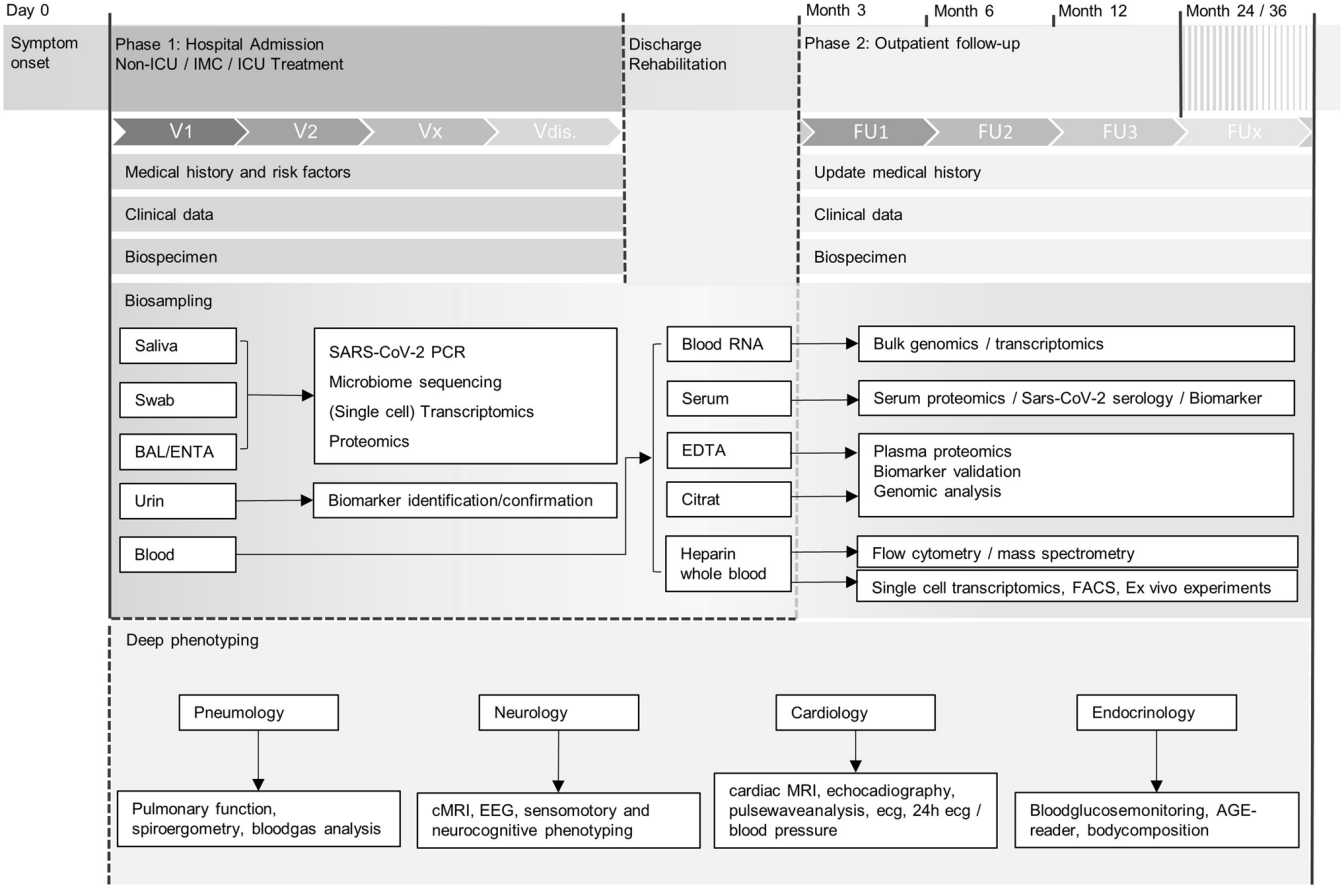
NAPKON-HAP study algorithm of data sampling and biosampling and deep phenotyping of the follow-up period: Upon hospital admission, participants are included, given SARS-CoV-2 infection and informed consent. Three study visits/week with biosampling are performed for 2 weeks for non-ICU and for 5 weeks for IMC/ICU patients during hospital admission. Biosampling starts at day of admission and will be continued at follow-up. Two deep phenotyping visits will take place at months 3 and 12 post-COVID-19 with a concise characterization of patients. Month 6 follow-up visits focus on pulmonary function testing. *ICU* intensive care unit, *IMC* intermediate care unit, *V* visit, *FACS* fluorescence activated cell sorting, *EDTA* ethylene diamine tetraacetic acid, *BAL* bronchoalveolar lavage, *ENTA* endotracheal aspirate, *FU* follow-up, *ECG* electrocardiogram, *AGE* advanced glycation end products, *MRI* magnetic resonance imaging, *EEG* electroencephalogram

Following hospital admission, the first study visit upon study inclusion surveys epidemiological and demographic parameters, medical history and potential risk factors, current medication, assessment of clinical status, disease symptoms, and patient-reported outcome measures (PROMIS) [20, 21]. In-hospital visits take place three times a week for up to 2 weeks in case of non-intensive care unit (ICU) treatment, and up to 5 weeks for ICU and intermediate care (IMC) unit treated patients. Blood sampling is performed during each visit, whereas sampling of urine, saliva, and oropharyngeal swabs are performed once a week. Data on disease severity as reflected by WHO ordinal scale for clinical improvement, concomitant medication, intercurrent diagnoses, and outcome are collected daily. At follow-up visits, laboratory blood testing and biosampling are continued (see Fig. 2, Table 1).

**Table 1:** NAPKON-HAP study schedule for the acute and follow-up phase of patients with COVID-19: time points of clinical follow-up visits, biospecimen collection, and assessment of patient-reported outcome and quality of life measures

Visit	V1-enrollment	V2	V3	V4	V5	V6	V7	In-hospital treatment			Outpatient follow-up					
								V8-V16	V17-Vxx	V-discharge	V9	V10	V11	V12	V13	UVx
Examination																
Visit schedule	Day of enrollment	Monday/Wednesday/Friday								Day of discharge	3-months deep phenotyping	6 months	12-months deep phenotyping	24 months	36 months	Unscheduled
Informed consent/authorization	X															
Clinical assessment																
Epidemiological and demographical data	X															
Medical history/update of medical history	X										X	X	X	X	X	X
Vital signs	X	X	X	X	X	X	X	X	X	X	X	X	X	X	X	X
Clinical symptoms	X	X	X	X	X	X	X	X	X	X	X	X	X	X	X	X
Physical examination											X	X	X	X	X	X
Routine blood testing	X	X	X	X	X	X	X	X	X	X	X	X	X	X	X	X
Recording of SARS-CoV-2 PCR testing	X	X	X	X	X	X	X	X	X	X						
Biobanking of serial blood samples	X	X	X	X	X	X	X	(X)	(X)		X	X	X	X	X	(X)
Biobanking of saliva and urine	X			X			X	(X)	(X)							
Documentation of concomitant medication	X	X	X	X	X	X	X	X	X	X	X	X	X	X	X	X
Documentation of clinical records from patient charts (parameters of mechanical ventilation, hemodynamics)	X	X	X	X	X	X	X	X	X	X						
Additional clinical data collection (chest X-ray, CT, echocardiography)	X	X	X	X	X	X	X	X	X	X	X	X	X	X	X	X
Record of any additional medical events, hospitalizations or ER visits	X	X	X	X	X	X	X	X	X	X	X	X	X	X	X	X
Clinical severity scores (GCS, SOFA)	X	X	X	X	X	X	X	X	X	X						
WHO clinical ordinal scale		Daily documentation during hospital stay								X	X	X	X	X	X	X
Scores and quality of life																
NYHA, PCFS, elements of rose NIHSS, frailty score, Barthel index, Katz index											X	X	X	X	X	X
Promis, Eq. 5D5L, MNSI, PDQ, NEI VFQ, SGRQ	X									X	X		X	X	X	X
Cardiology follow-up																
ECG, echocardiography										X	X	X	X	X	X	X
24 h ECG and blood pressure, cardiac MRI, pulse wave analysis											X		X	X	X	X
Respiratory follow-up																
Body plethysmography, diffusion capacity, respiratory muscle function test, blood gas analysis										X	X	X	X	X	X	X
Cardiopulmonary exercise testing											X		X	X	X	X
Neurology/psychiatry/ophthalmology follow-up																
Physical activity testing, MoCA, CANTAB											X		X	X	X	X
Brain MRI, EEG, somatosensory testing, smell/taste test											X		X	X	X	X
Optical coherence tomography, funduscopy /photography											X		X	X	X	X
Endocrinology follow-up																
Advanced glycation end products, body composition, glucose monitoring											X		X	X	X	X

Cardiopulmonary, neuropsychiatric, and endocrine follow-up visits start within 3 months post-SARS-CoV-2 infection, followed by 6 and 12 month follow-up visits. In case of pathological findings, those findings will be surveyed up to 36 months

Harmonized collection of biospecimens in participating study sites was initially orchestrated by the NAPKON biosample core unit technically supported by the Laboratory Information Management System (LIMS) of German Center for Cardiovascular Research (DZHK), and has recently been transferred to the NAPKON NUKLEUS infrastructure [22]. The aim is to ensure consistent collection, processing, storage, and documentation of biospecimens and to enable usability of biospecimens collected within NAPKON-HAP. Regular monitoring visits will ensure high standards in biospecimen quality according to the Handbook for Quality Management in Biobanking [23].

Biosampling of the respiratory tract includes saliva and nasopharyngeal swabs per protocol from all patients as well as aliquots of bronchoalveolar lavage fluid (BAL) and endotracheal aspirate (ENTA) obtained within standard of care in case of invasively ventilated patients. Blood sampling includes serum, plasma, PBMCs, heparinized whole blood, and blood RNA for subsequent genotyping, transcriptomic and proteomic analyses, flow cytometry, and mass spectrometry as well as serum analysis of biomarkers and serological testing of antibodies and cytokines (see Fig. 2).

Deep phenotyping at months 3 and 12 include detailed pulmonary testing (including—but not restricted to—body plethysmography, pulmonary muscle strength testing, and spiroergometry), cardiological examination (cardiac magnetic resonance imaging (MRI), echocardiography, pulse wave analysis), neurological exploration (brain MRI, electroencephalogram (EEG), somatosensory testing), and endocrine investigation (14 days subcutaneous glucose monitoring). A comprehensive list of investigations is given in Table 1. Only in case of pathological findings, the latter examinations are performed at follow-up visits at months 24 and 36.

### Harmonized data collection in electronic case report form (eCRF)

Data collection and biosampling are performed in accordance with the WHO supported case report form (CRF) proposed by the International Severe Acute Respiratory and Emerging Infection Consortium (ISARIC) [24]. Items of the ISARIC-CRF were translated into German language using the standardized functional assessment of chronic illness therapy (FACIT) translation methodology [25]. The parameters were selected and adapted to local standards through a multidisciplinary expert review board [18]. All collected data at study sites including source documents and laboratory reports are being documented in eCRF. Medical and research records for this study are maintained in compliance with International Conference on Harmonization guideline for Good Clinical Practice (ICH-GCP) [26].

### Data management and storage

The data management and study database are located at the Institute for Medical Informatics of the University Medical Center Göttingen. To capture data, the GCP-compliant software secuTrial® is used.

Patient identifying data are kept at the study sites and securely stored separately at the University Medical Center Greifswald (Zentrale Daten-Treuhandstelle, https://www.ths-greifswald.de/en), where pseudonyms are generated and provided for data-, biomaterial- and image storage [27]. Integration of existing data and biospecimen of established clinical COVID-19 cohorts within Germany into the NAPKON-HAP platform was successfully performed and reviewed by an independent board.

### Image data management and storage

Infrastructure for pseudonymized storage of image data is provided by the TrialComplete system. It enables pseudonymized DICOM data upload and transfer from the study centers to central imaging labs. eCRF data are automatically synchronized between the data management system and the image data storage system.

### Data access and sharing

NAPKON established a core unit for overall coordination and interaction with scientists and partner sites. It implements and governs the use and access committee (UAC). The UAC steers user requests and decides upon the use of clinical data and biosamples for scientific projects. NAPKON-HAP aims to provide the research community with data and biosamples for their projects in a non-bureaucratic manner at the same time safeguarding patients’ rights. To date, 133 research projects are registered using NAPKON-HAP data.

### Ethics and registration

The principles of Good Clinical Practice and other applicable regulations and guidelines are used to guide procedures and considerations. The study protocol and its amendments were reviewed and approved by the Charité Ethics Committee (EA2/066/20, EA2/226/21) as well as local ethics committees at each participating study center.

## Conclusion

Within the first 2 years of the COVD-19 pandemic, academia has successfully demonstrated how to spark basic research on a novel pathogen and the host–pathogen interaction. In Germany, the network of medical universities was established in 2020 with the support of the German Federal Ministry of Education and Research, aiming to provide a research infrastructure for the COVID-19 and potential future pandemics. Clinical research was concerted within three observational cohort study platforms within the National Pandemic Cohort Network built on common core infrastructure units. Here, we describe the study protocol of the high-resolution platform, a multi-centered observational cohort study of patients hospitalized in one of the ten participating medical university centers with SARS-CoV-2 infection or non-COVID community acquired pneumonia or ARDS as controls. The study protocol expands from disease onset until 36 months follow-up and comprises a harmonized collection of clinical data as well as standardized biosampling procedures of blood and respiratory tract specimens. Data and biosamples are stored centrally and available for researchers through a use and access process after real-time verification of the status of consent documents. Exceptional clinical and biological data and specimen are especially designed for deep phenotyping projects including transcriptomics, proteomics, epigenomics, and metabolomics and will enhance translational research of COVID-19 and long COVID multi-omic approaches.

With this study, we will add significant scientific insights and provide high-quality data and biospecimen to aid researchers to investigate COVID-19 pathophysiology, pathology, and chronic morbidity.

## Data Availability

Not applicable.

## References

[1] Wu F (2020). A new coronavirus associated with human respiratory disease in China. Nature.

[2] Zhou P (2020). A pneumonia outbreak associated with a new coronavirus of probable bat origin. Nature.

[3] WHO. WHO Coronavirus Disease (COVID-19) Dashboard. https://covid19.who.int/.

[4] ECDC. European Centre for Disease Prevention and Control Situation updates on COVID-19. 2020. https://www.ecdc.europa.eu/en/covid-19/situation-updates. 10.2807/1560-7917.ES.2020.25.6.2002131 PMC7029450 32070466

[5] Johns Hopkins University Coronavirus Resource Center. 2020. https://coronavirus.jhu.edu/.

[6] Kousha K, Thelwall M (2020). COVID-19 publications: database coverage, citations, readers, tweets, news, Facebook walls, Reddit posts. Quant Sci Stud.

[7] Dexamethasone in Hospitalized Patients with Covid-19—Preliminary Report. N Engl J Med. 2020. 10.1056/NEJMoa2021436. 10.1056/NEJMoa2021436 PMC7383595 32678530

[8] Pan H et al., Repurposed antiviral drugs for COVID-19—interim WHO SOLIDARITY trial results. medRxiv. 2020:2020.10.15.20209817. 10.1101/2020.10.15.20209817. 10.1056/NEJMoa2023184 PMC7727327 33264556

[9] Polack FP (2020). Safety and efficacy of the BNT162b2 mRNA Covid-19 vaccine. N Engl J Med.

[10] Baden LR (2020). Efficacy and safety of the mRNA-1273 SARS-CoV-2 vaccine. N Engl J Med.

[11] Team W, WHO Guideline Development Group for Clinical Management of COVID-19 (V3). 2020:62. WHO/2019-nCoV/clinical/2020.5.

[12] Knight SR (2020). Risk stratification of patients admitted to hospital with covid-19 using the ISARIC WHO clinical characterisation protocol: development and validation of the 4C Mortality Score. BMJ.

[13] Wiersinga WJ (2020). Pathophysiology, transmission, diagnosis, and treatment of coronavirus disease 2019 (COVID-19): a review. JAMA.

[14] Leisman DE (2020). Cytokine elevation in severe and critical COVID-19: a rapid systematic review, meta-analysis, and comparison with other inflammatory syndromes. Lancet Respir Med.

[15] Nishiga M (2020). COVID-19 and cardiovascular disease: from basic mechanisms to clinical perspectives. Nat Rev Cardiol.

[16] Pérez CA. Looking ahead. The risk of neurologic complications due to COVID-19. 2020;**10**: 371–374. 10.1212/cpj.0000000000000836. 10.1212/CPJ.0000000000000836 PMC7508336 32983618

[17] Sass J, et al. The German Corona Consensus Dataset (GECCO): a standardized dataset for COVID-19 research. medRxiv. 2020:2020.07.27.20162636. 10.1101/2020.07.27.20162636. 10.1186/s12911-020-01374-w PMC7751265 33349259

[18] Kurth F (2020). Studying the pathophysiology of coronavirus disease 2019: a protocol for the Berlin prospective COVID-19 patient cohort (Pa-COVID-19). Infection.

[19] Dunning JW (2014). Open source clinical science for emerging infections. Lancet Infect Dis.

[20] Alonso J (2013). The case for an international patient-reported outcomes measurement information system (PROMIS^®^) initiative. Health Qual Life Outcomes.

[21] Cella D (2007). The Patient-Reported Outcomes Measurement Information System (PROMIS): progress of an NIH Roadmap cooperative group during its first two years. Med Care.

[22] GBM. German Biobanc Node. https://www.bbmri.de/.

[23] Sabrina SB, Meinung Karl-Friedrich, Becker Julia, Slotta-Huspenina Michael, Kiehntopf Peter, Schirmacher Christiane, Hartfeldt Esther, Herpel Michael, Hummel, German Biobank Node: Handbook for Quality Management in Biobanking. 2020: Zenodo. 10.5281/zenodo.1420472.

[24] WHO I, ISARIC/WHO Clinical Characterisation Protocol for Severe Emerging Infections. 2014.

[25] FACIT. Functional Assessment of Chronic Illness Therapy. https://www.facit.org/.

[26] ICH harmonised guideline integrated addendum to ICH E6(R1): guideline for good clinical practice E6(R2). 2016.

[27] Zentrale Daten-Treuhandstelle. https://www.medizin.uni-greifswald.de/ru/forschung-lehre/core-units/treuhandstelle/.

